# Wendan decoction for primary insomnia: Protocol for a systematic review and meta-analysis

**DOI:** 10.1097/MD.0000000000008906

**Published:** 2017-11-27

**Authors:** Xia Yan, Yuanping Wang, Xiaohui Li, Ziqin Li, Yu Zhang, Xiaoli Cai, Dawei Wang

**Affiliations:** a 2nd Affiliated Hospital of Guangzhou University of Chinese Medicine, Guangzhou; b Guangdong Provincial Key Laboratory of Research on Emergency in TCM; c Guangzhou University of Chinese Medicine, Guangzhou, China.

**Keywords:** primary insomnia, protocol, systematic review, Wendan decoction

## Abstract

**Background:**

Insomnia is a widespread complaint in the general population. Wendan decoction has been widely applied in the treatment of primary insomnia. However, to our knowledge, there has been no systematic review or meta-analysis of randomized controlled trails regarding the effectiveness of this treatment. Here, we provide a protocol to evaluate the efficacy and safety of Wendan decoction for primary insomnia.

**Methods and analysis:**

Relevant randomized controlled trials in 5 English databases [EMBASE, the Cochrane Central Register of Controlled Trials (Cochrane Library), PubMed, the Allied and Complementary Medicine Databases (AMED), and the Cumulative Index to Nursing and Allied Health Literature (CINAHL)], 4 Chinese databases [Chinese Biomedical Literature Database (CBM), Chinese Medical Current Content (CMCC), China National Knowledge Infrastructure (CNKI), and Wanfang Database] will be comprehensively searched by 2 researchers in October 2017. The therapeutic effects according to the Pittsburgh Sleep Quality Index (PSQI) will be accepted as the primary outcomes. We will use RevMan V.5.3 software as well to compute the data synthesis carefully when a metaanalysis is allowed.

**Results:**

This study will provide a high-quality synthesis of current evidence of Wendan decoction for primary insomnia from several aspects including PSQI, the total scores of the Insomnia Severity Index (ISI), syndrome according to standards for assessing Traditional Chinese medicine and adverse events.

**Conclusion:**

The conclusion of our systematic review will provide evidence to judge whether Wendan decoction is an effective intervention for patient with primary insomnia.

**Ethics and dissemination:**

The outcomes of this systematic review will offer implications of the use of Wendan decoction treatment for primary insomnia patients. This knowledge informing recommendations will be provided by researchers who are interested in the treatment of primary insomnia. The results of this study will be disseminated through presentation at a conference and publication of the data in a peer-reviewed journal.

**PROSPERO registration number:**

PROSPERO CRD 42017065664.

## Abstract 2

Supplemental Digital Content is available in the text

## 1 Introduction

Insomnia is one of the most common sleep disorders. Six percent of the adults meet the diagnostic criteria of insomnia and suffer from a chronic condition. For nearly half of the patients with severe insomnia, the course would last more than 10 years.^[1]^ Insomnia has become an important factor to affect the health and quality of life modern people.^[2]^ It can even cause contingencies that would endanger personal and public safety, such as traffic accidents.^[3]^ All these increase heavy burdens for both individuals and society. In addition, recent studies have found that insomnia is also a risk factor for various cardiovascular and cerebrovascular diseases and requires highest attention.^[4,5]^

Currently, the treatments for insomnia include psychotherapy, medication, physical therapy, etc., among which the psychological and behavioral therapy are preferred.^[6,7]^ Cognitive behavioral therapy for insomnia (CBTI), the most common treatment, is effective to improve the subjective sleep results.^[8]^ However, it has not been fully applied in clinical practice due to poor patient compliance and lack of trained psychotherapists.^[9]^ Drugs such as benzodiazepine receptor agonists (BZRAs) are thought to be beneficial for improving sleep.^[10]^ Yet there are potential risks, including addiction as a result of long-term usage.^[11]^ Hence, there are increasingly more insomniacs who are seeking complementary and alternative therapies.^[12]^

Traditional Chinese medicine (TCM) recorded at first in ancient China is a part of complementary and alternative medicine, and has been extensively used in curing primary insomnia patients for more than 2000 years.^[13]^ Nowadays, TCM is increasingly popular among insomnia individuals around the world, including Chinese herbal medicine (CHM), qigong, acupuncture, and moxibustion.^[14,15]^ Two published meta-analyses drew a conclusion that CHM was more effective than BZRAs and placebo in the treatment of primary insomnia, and less likely to have adverse effects.^[16,17]^

Wendan decoction (WDD), a famous CHM prescription, was recorded originally in an ancient book named *Prescriptions Worth a Thousand Gold,* by Simiao Sun in 652 AD, and is widely used in the treatment of mental disorders, especially in improving the condition of poor sleep.^[16]^ WDD is comprised of 6 kinds of CHMs: Banxia (Pinellia tuber), Zhishi (Immature orange fruit), Zhuru (bamboo shavings), Fuling (Indian bread), Chenpi (Dried tangerine peel), and Gancao (Liquorice root), all of which are standardly marked in Chinese Pharmacopoeia (V.2015). According to Wu et al,^[18]^ WDD can also be applied to improve the insomnia-related depression through adjusting sleep-deprived induced destructive sentiments by regulating orexin-A and leptin expression, which in turn may defend against insomnia.

However, to our knowledge, few systematic reviews have been conducted to evaluate the effectiveness and safety of WDD for primary insomnia. Through the review, we aim to answer 2 clinical questions regarding primary insomnia: whether WDD is more remarkably curative than conventional medication or placebo, and whether WDD is safer with less adverse effects compared with traditional therapy for primary insomnia.

## 2 Methods

### 2.1 Inclusion criteria for study selection

#### 2.1.1 Types of studies

All the randomized controlled trials (RCTs) of WDD for the management of primary insomnia patients will be included without writing language or publication status restriction.

#### 2.1.2 Types of patients

Participants with primary insomnia will be included without the restrictions of age, gender, or ethnic background. Primary insomnia must be diagnosed according to the Diagnostic and Statistical Manual of Mental Disorders-Fourth Edition criteria^[19]^ and Chinese Classification and Diagnosis of Mental Disease.^[20]^

#### 2.1.3 Types of interventions

The therapy used in the experimental group is a prescription including the main ingredients of WDD, which is taken orally. The dosage form can be decoction, granules, or other modern dosage forms. The dosage and treatment period are not limited. The control group could be blank control, placebo, conventional western medicine (such as benzodiazepines), or psychological intervention. If combined treatment of traditional Chinese and western medicine is given, the treatment of western medicine in the control group should be consistent with that of the experimental group.

#### 2.1.4 Types of outcome measures

##### 2.1.4.1 Primary outcomes

Sleep quality will be evaluated with the Pittsburgh Sleep Quality Index (PSQI) as the primary outcome.^[21]^

##### 2.1.4.2 Secondary outcomes

Secondary outcomes will include the total scores of the Insomnia Severity Index (ISI)^[22]^; syndrome according to standards for assessing TCM; and adverse events, such as nausea, dizziness, vomiting, and fatigue.

### 2.2 Search methods for the identification of studies

Five English databases [EMBASE, PubMed, the Cochrane Central Register of Controlled Trials (Cochrane Library), the Allied and Complementary Medicine Databases (AMED), and the Cumulative Index to Nursing and Allied Health Literature (CINAHL)] and 4 Chinese databases [Chinese Biomedical Literature Database (CBM), China National Knowledge Infrastructure (CNKI), Chinese Medical Current Content (CMCC), and Wanfang Database] will be comprehensively searched on October 2017 for the RCTs regarding WDD for primary insomnia. Search terms will be as follows: insomnia, WDD, and RCTs. The search terms with the same English meaning will be also used in Chinese databases. The detailed strategies for searching the PubMed database will be presented in Appendix A and modified by using other databases.

#### 2.2.1 Searching other resources

Relevant systematic review or meta-analysis of RCTs will be search electronically. In addition, relevant conference proceedings, references list of eligible studies, and gray literatures will also be manually searched for further resources.

### 2.3 Data collection and analysis

#### 2.3.1 Selection of studies

**Figure 1:**
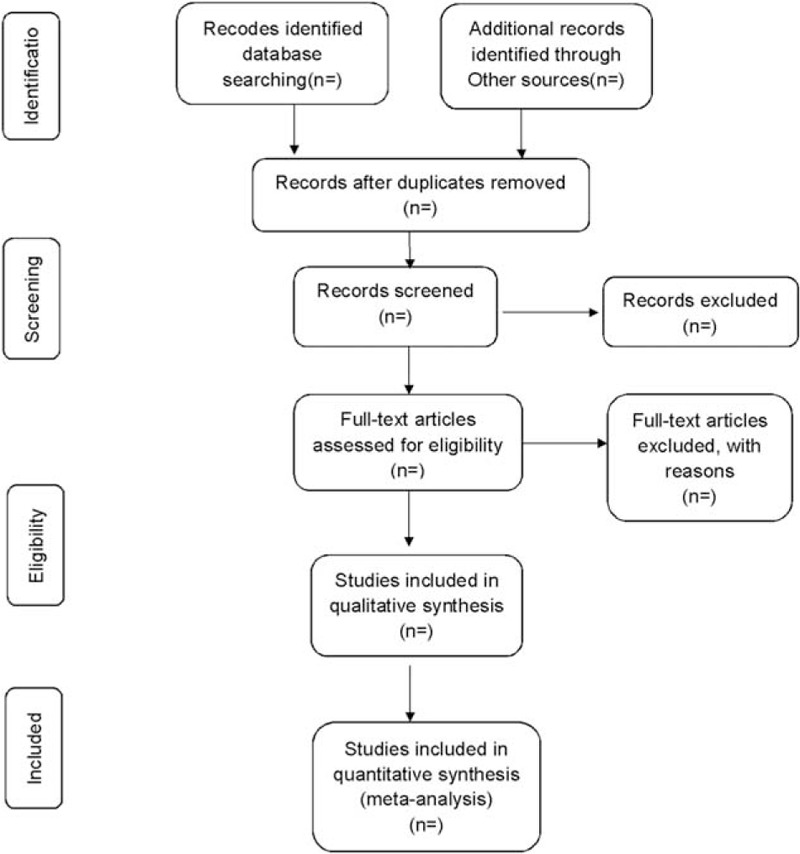
Flow diagram of study selection process.

The corresponding research members will import the retrieved articles into the literature management system of EndnoteX7 and remove repetitive data. Later, the obvious disqualified literatures will be excluded by the title and the abstract. The final literature will then be determined after reading the whole articles, group discussion, and contacting the author to learn about the details of the study (Fig. 1). The final list of documents is translated into Microsoft Excel format. Both the literature search and the literature screening will be carried out independently by 2 researchers. Eventually, another member in the research team will work out the inconsistencies and check the final included literature.

#### 2.3.2 Data extraction and management

Two of the review authors will independently scan the full texts of each article to extract the data via a standardized data abstraction form. Basic general information will be extracted, including authors, year of publication, age, gender, disease duration, the duration of follow-up, sample size, details of intervention, and control. Outcome and further information including results, adverse events, and conflicts of interest will be extracted as well. Any disagreements will be discussed and judged between 2 reviewers. The final results of the extraction and further disagreements will be checked and arbitrated by the third reviewer.

#### 2.3.3 Assessment of risk of bias in included studies

The risk of bias of the included articles will be evaluated by 3 independent raters according to the tool recommended by Cochrane Handbook V.5.1.0, which consists of the following 7 domains: random sequence generation, allocation concealment, blinding of participants, personnel and outcome, incomplete outcome data addressed, selective reporting, and other bias. The quality of the reporting will be categorized into 3 levels: low risk of bias, unclear risk of bias, or high risk of bias. Discrepancies will be discussed among the 3 raters to come to a consensus. If necessary, a senior reviewer will be consulted.

#### 2.3.4 Measures of treatment effect

For continuous data, the extracted data will be evaluated using a standard mean difference (SMD) with 95% confidence interval (95% CI). For dichotomous outcomes, a rate ratio (RR) with 95% CI will be presented for analysis.

#### 2.3.5 Dealing with missing data

We will try to correspond with the first author to request the missing or insufficient trial data. If we are unable to obtain the missing data, only the available data and potential impact of the missing data will be analyzed in the discussion.

#### 2.3.6 Assessment of heterogeneity

The *I*^2^ statistic will be performed for the quantification of inconsistencies among the included trials. If an *I*^2^ value exceeds 50%, the statistic heterogeneity among trials will be considered significant. Further subgroup analysis will be conducted to investigate the potential causes of heterogeneity.

#### 2.3.7 Assessment of reporting bias

If sufficient studies are involved in the review (more than 10 trials), we will generate visual asymmetry on a funnel plot using Egger methods, which aims to detect reporting biases. Regarding the methodological quality, all the eligible trials will be included.

#### 2.3.8 Data synthesis

RevMan software (Version 5.3, Copenhagen: The Nordic Cochrane Center, The Cochrane Collaboration, 2014) will be used to compute the data synthesis carefully when sufficient evidence proves that a meta-analysis is suitable. The fixed-effects model will be applied for data synthesis with low heterogeneity (*I*^2^ < 50%). If not, the random-effects model will be conducted.

#### 2.3.9 Subgroup analysis

According to different control interventions, inconsistent participants characteristic, and outcome measures, subgroup analysis will be performed if the included studies are sufficient (at least 10 trials). The aim of the subgroup analysis is to explore the resources of the heterogeneity.

#### 2.3.10 Sensitivity analysis

We will conduct sensitivity analysis to identify the quality of studies according to the following: sample size; the effect of missing data; and methodological quality.^[23]^

#### 2.3.11 Grading the quality of evidence

We will evaluate the quality of evidence by the Grading of Recommendations Assessment, Development and Evaluation (GRADE) and rate it into high, moderate, low, or very low 4 levels.^[24]^

## 3 Discussion

**Figure 2:**
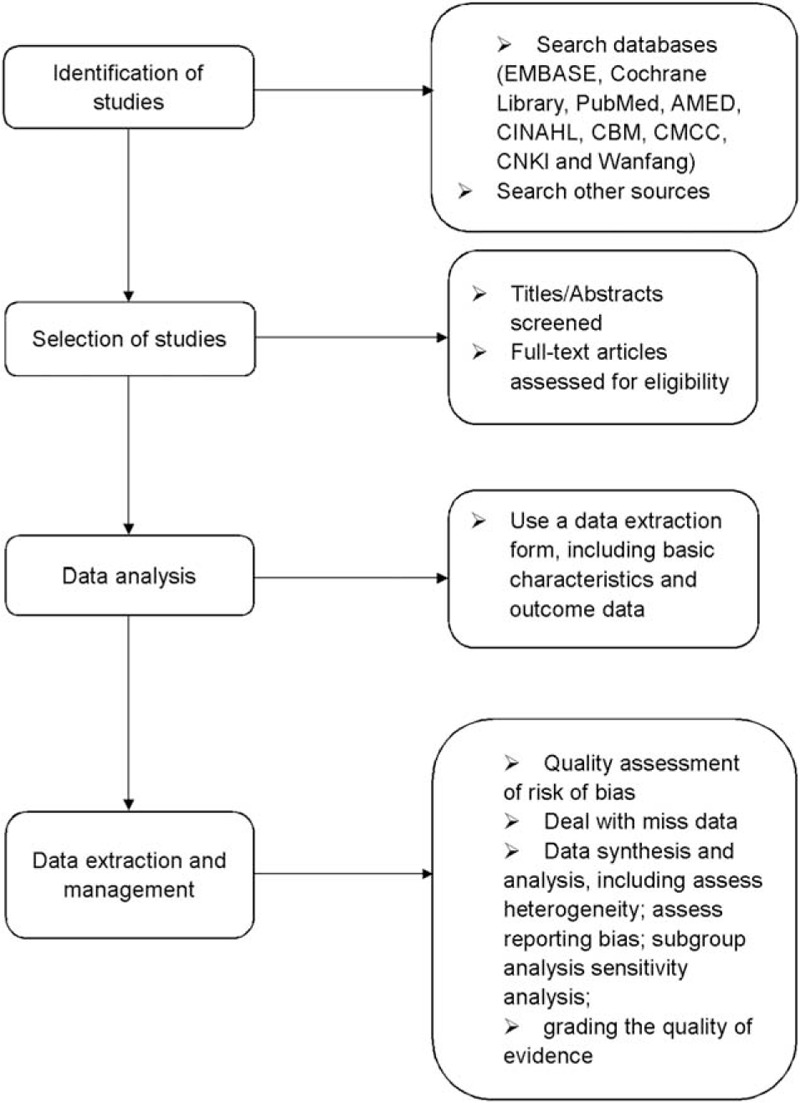
Flow diagram of the systematic review and meta-analysis.

First-line drugs for insomnia include benzodiazepines and BZRAs.^[25]^ However, these drugs are associated with a number of undesirable side effects of long-term treatment such as increasing the risk of accidents^[26]^ and the rate of mortality.^[27]^ WDD may be a useful treatment for primary insomnia, and it is unlikely to produce severe side effects. To our knowledge, whether WDD can improve the condition of patients with primary insomnia or not has not been clearly stated. Therefore, a high-quality systematic review and meta-analysis is necessary, and the process can be shown in the flow diagram (Fig. 2). It is expected that this review will provide more objective evidences of WDD for primary insomnia. However, there are some limitations in this systematic review. First, the included trails are limited to the publication of Chinese or English, which may lead to selection bias. Second, different dosage of herbal medicine, age of patients, and degree of insomnia severity may run risk of heterogeneity. Finally, the investigation that contains small sample of included trials may lead to the high risk of bias.

PRISMA-P (Preferred Reporting Items for Systematic review and Meta-Analysis Protocols) checklist of this protocol is presented in online supplementary.

## Supplementary Material

Supplemental Digital Content
